# Impaired Object Recognition but Normal Social Behavior and Ultrasonic Communication in Cofilin1 Mutant Mice

**DOI:** 10.3389/fnbeh.2018.00025

**Published:** 2018-02-19

**Authors:** A. Özge Sungur, Lea Stemmler, Markus Wöhr, Marco B. Rust

**Affiliations:** ^1^Molecular Neurobiology Group, Institute of Physiological Chemistry, Philipps-University of Marburg, Marburg, Germany; ^2^Department of Behavioral Neuroscience, Experimental and Biological Psychology, Philipps-University of Marburg, Marburg, Germany; ^3^Marburg Center for Mind, Brain and Behavior (MCMBB), Philipps-University of Marburg, Marburg, Germany; ^4^DFG Research Training Group—Membrane Plasticity in Tissue Development and Remodeling, Philipps-University of Marburg, Marburg, Germany

**Keywords:** USV, memory, cognition, ADF/cofilin, actin depolymerizing factor, dendritic spine

## Abstract

Autism spectrum disorder (ASD), schizophrenia (SCZ) and intellectual disability (ID) show a remarkable overlap in symptoms, including impairments in cognition, social behavior and communication. Human genetic studies revealed an enrichment of mutations in actin-related genes for these disorders, and some of the strongest candidate genes control actin dynamics. These findings led to the hypotheses: (i) that ASD, SCZ and ID share common disease mechanisms; and (ii) that, at least in a subgroup of affected individuals, defects in the actin cytoskeleton cause or contribute to their pathologies. Cofilin1 emerged as a key regulator of actin dynamics and we previously demonstrated its critical role for synaptic plasticity and associative learning. Notably, recent studies revealed an over-activation of cofilin1 in mutant mice displaying ASD- or SCZ-like behavioral phenotypes, suggesting that dysregulated cofilin1-dependent actin dynamics contribute to their behavioral abnormalities, such as deficits in social behavior. These findings let us hypothesize: (i) that, apart from cognitive impairments, cofilin1 mutants display additional behavioral deficits with relevance to ASD or SCZ; and (ii) that our cofilin1 mutants represent a valuable tool to study the underlying disease mechanisms. To test our hypotheses, we compared social behavior and ultrasonic communication of juvenile mutants to control littermates, and we did not obtain evidence for impaired direct reciprocal social interaction, social approach or social memory. Moreover, concomitant emission of ultrasonic vocalizations was not affected and time-locked to social activity, supporting the notion that ultrasonic vocalizations serve a pro-social communicative function as social contact calls maintaining social proximity. Finally, cofilin1 mutants did not display abnormal repetitive behaviors. Instead, they performed weaker in novel object recognition, thereby demonstrating that cofilin1 is relevant not only for associative learning, but also for “non-matching-to-sample” learning. Here we report the absence of an ASD- or a SCZ-like phenotype in cofilin1 mutants, and we conclude that cofilin1 is relevant specifically for non-social cognition.

## Introduction

Autism-spectrum disorder (ASD), schizophrenia (SCZ) and intellectual disability (ID) are neuropsychiatric disorders with high heritability that show a remarkable overlap in behavioral symptoms. For instance, cognitive deficits are present in all three disorders and social or communication impairments have been reported for ASD and SCZ (Abrahams and Geschwind, 2008; Fung et al., 2012; Millan et al., 2012; Volkmar and McPartland, 2014; Pasciuto et al., 2015). While the underlying disease mechanisms largely remained unknown, the overlap in behavioral symptoms suggested shared pathomechanisms for ASD, SCZ and ID.

Human genetic analyses revealed an enrichment of mutations in genes regulating actin filaments (F-actin) at glutamatergic synapses for all three neuropsychiatric disorders (Ramakers, 2002; Gilman et al., 2011; Fromer et al., 2014). Moreover, some of the strongest candidate genes known to be involved modulate assembly or disassembly of F-actin (Reeve et al., 2005; Durand et al., 2012; Duffney et al., 2013, 2015; Steinecke et al., 2014; Peykov et al., 2015). Exemplarily, mutations in *Shank3*, a gene which encodes for a postsynaptic scaffolding protein at glutamatergic synapses, constitute a highly prevalent and penetrant risk factor for ASD (Monteiro and Feng, 2017), and genes encoding for members of the 14-3-3 protein family are risk genes for SCZ (Muratake et al., 1996; Toyooka et al., 1999; Bell et al., 2000; Wong et al., 2005). These findings moved actin regulatory proteins into the focus as critical regulators of brain function and behavior, and led to the hypothesis that dysregulation of synaptic actin dynamics may be a common pathophysiological condition for ASD, SCZ and ID (Spence and Soderling, 2015). In support of this hypothesis, several mutant mouse strains with targeted inactivation of actin regulatory proteins displayed behavioral symptoms reminiscent of these neuropsychiatric disorders (Meng et al., 2002; Soderling et al., 2007; Peleg et al., 2010; Rust et al., 2010; Carlson et al., 2011; Kim et al., 2015).

Actin depolymerizing factor (ADF)/cofilin proteins are essential regulators of actin dynamics that promote F-actin disassembly by accelerating the dissociation of actin subunits and by severing F-actin (for review Kanellos and Frame, 2016). Although all three family members are expressed in the brain (Bellenchi et al., 2007; Görlich et al., 2011; Gurniak et al., 2014), cofilin1 emerged as the predominant regulator of synaptic actin dynamics (for review Rust, 2015). Specifically, several studies demonstrated an important function for cofilin1 in activity-induced changes of both dendritic spine morphology and postsynaptic accumulation of glutamate receptors (Gu et al., 2010; Hotulainen and Hoogenraad, 2010; Rust et al., 2010; Bosch et al., 2014). Accordingly, by exploiting gene-targeted mice lacking cofilin1 in excitatory neurons of the postnatal brain, we found that cofilin1 specifically controls postsynaptic plasticity and that disturbed postsynaptic plasticity impaired their performance in paradigms of associative learning such as Morris water maze, conditional place preference or contextual and cued fear conditioning (Rust et al., 2010). In line with our findings, dysregulation of ADF/cofilin activity is believed to contribute to ID pathology in humans (Frangiskakis et al., 1996; Spence and Soderling, 2015; Pyronneau et al., 2017; Vogel Ciernia et al., 2017). Notably, recent studies in mutant mice implied a role for cofilin1-dependent actin dynamics in ASD and SCZ pathologies. Specifically, cofilin1 over-activation has been associated with both: (i) ASD-like behavioral deficits including impaired social approach and abnormal self-grooming activity in *Shank3* mutant mice; and (ii) SCZ-like behavioral deficits including hyper-locomotion and impaired working memory, social approach and social recognition in 14-3-3 functional knockout (FKO) mice (Duffney et al., 2015; Foote et al., 2015). Based on these findings, we hypothesized the presence of additional behavioral deficits in cofilin1 mutant mice and that these mutants may present a novel mouse model for ASD and/or SCZ. To test our hypothesis, we compared juvenile cofilin1 mutants to control littermates and comprehensively characterized social behavior by determining direct reciprocal social interaction, social approach and social memory. In none of these paradigms did we find obvious deficits, suggesting that cofilin1 is dispensable for social behavior. Further, cofilin1 mutants did not show any ultrasonic communication deficits or repetitive behavior during reciprocal social interaction. Instead, they performed weaker in novel object recognition, thereby confirming that cofilin1 is crucial for non-social cognition. Together, cofilin1 mutants displayed cognitive deficits, but no other behavioral phenotypes with relevance to ASD or SCZ core symptoms, such as impaired social behavior, impaired ultrasonic communication or repetitive behavior. We therefore conclude that cofilin1 is relevant specifically for non-social cognition.

## Materials and Methods

### Animals and Housing

As previously described (Rust et al., 2010), mice with a specific deletion of cofilin1 (non-muscle cofilin, n-cofilin) in excitatory neurons of the postnatal telencephalon were generated by exploiting conditional cofilin1 mice (Cfl1^flx/flx^) and transgenic mice expressing Cre recombinase under control of the CaM-kinase II α-subunit promoter (CaMKII-cre; Minichiello et al., 1999; Bellenchi et al., 2007). For our experiments, cofilin1 mutants (Cfl1^flx/flx, CaMKII-cre^, termed KO throughout the manuscript) and control littermates (Cfl1^flx/flx^, termed CTR) were obtained by breeding of a Cfl1^flx/flx^ male with a Cfl1^flx/flx, CaMKII-cre^ female or a Cfl1^flx/flx^ female with a Cfl1^flx/flx, CaMKII-cre^ male, respectively. Mice were bred in conventional vivariums in the animal facility of the Philipps-University of Marburg, Germany. Approximately 2 weeks after breeding, females were individually housed and inspected daily for pregnancy and delivery. The day of birth was considered as postnatal day (PND) 0. Offspring were identified by earmarks at PND14, and they were genotyped as previously described (Rust et al., 2010). After weaning on PND21, mice were socially housed in groups of 2–5 with same-sex partners in polycarbonate Makrolon type II or type III cages (Ehret, Emmendingen, Germany). Bedding and paper tissue were provided to each cage. Standard rodent chow and water were available *ad libitum*. The colony room was maintained on a 12:12 light/dark cycle (lights on at 06:00 am) at approximately 22°C and 40%–50% humidity. All experiments were carried out in accordance with the guidelines laid down by the European Community Council Directive of 24 November 1986 (86/609/EEC), and they have been approved by the ethical committee of the Regierungspräsidium Gießen, Germany (file reference: V54-19c2015h01MR20/30 Nr. G40/2016).

### Overview of Behavioral Procedures

Subject mice were tested in three social behavior paradigms: direct reciprocal social interaction for measuring social behavior and ultrasonic communication in freely-moving pairs, social approach for assessing social motivation, and social recognition for assessing social memory. In addition, a non-social memory paradigm, i.e., novel object recognition, was conducted. Reciprocal social interaction was tested between PND22–26. Social approach, social recognition and novel object recognition were performed between PND24–32. Social behavior assays and the non-social memory task were performed in a balanced order, with social approach and social recognition being performed on 1 day and novel object recognition the other day. All behavioral paradigms were performed under dim red light during the light phase of the 12:12 h-light-dark-cycle. Prior to each test, behavioral equipment was thoroughly cleaned using a 0.1% acetic acid solution followed by drying. Body weight was measured after behavioral testing. Experimenters were blind to genotypes during data acquisition and behavioral analyses.

### Reciprocal Social Interaction

To measure reciprocal social interaction behavior, pairs of juvenile mice were allowed to socially interact for 5 min after one mouse of the pair being habituated to the test environment for 1 min, using a previously established protocol (Wöhr et al., 2015). Only same-sex/same-genotype pairs consisting of non-littermates were used. To enhance the level of social motivation, juvenile mice were socially isolated for 24 h prior to testing. Testing was performed in a clean Makrolon type III cage with fresh bedding and a metal lid under dim red light. Behavior was recorded using a video camera placed 30 cm away from the cage. Interaction-induced ultrasonic vocalization (USV) emission was monitored by an UltraSoundGate Condenser CM 16 Microphone sensitive to frequencies of 15–180 kHz (flat frequency response between 25 kHz and 140 kHz; ±6 dB; Avisoft Bioacoustics, Berlin, Germany), placed 15 cm above the cage lid. The microphone was connected via an UltraSoundGate 416 USGH audio device (Avisoft Bioacoustics) to a personal computer, where acoustic data were recorded with a sampling rate of 250 kHz (16 bit) by Avisoft RECORDER (version 2.97).

### Social Approach, Social Recognition and Novel Object Recognition

Social approach, social recognition and novel object recognition were performed in a three-chambered box, similar to our previous studies (Sungur et al., 2017). The box was made of dark gray polycarbonate material and consisted of two side chambers (230 × 345 × 350 mm) connected through a smaller chamber (145 × 70 × 350 mm) located centrally between both side chambers. This middle chamber had two retractable doors to control access to the side chambers. Behavioral testing in the three-chambered box was conducted on three consecutive days. On the first day, subject mice were individually kept for 30 min in a Makrolon type III cage and were then allowed to explore the empty three-chambered box for 30 min in order to habituate to the apparatus. On the second and third day, subject mice were again placed in this chamber for 30 min. Subsequently, social behavior paradigms or non-social memory tasks were performed in a balanced order, with social approach and social recognition being performed on 1 day and novel object recognition on the other day.

#### Social Approach and Social Recognition

After being individually kept in a Makrolon type III cage for 30 min, subject mice were tested for social approach and social recognition (Nadler et al., 2004), using a modified protocol previously established (Sungur et al., 2017). Testing consisted of three phases, i.e., social approach trial (10 min), inter-trial interval (30 min), and social recognition trial (10 min). In the social approach trial, subject mice were allowed to freely explore for 10 min the three-chambered box containing an empty wired-cage (object, non-social stimulus) in one side chamber and a stimulus mouse (age- and sex-matched wildtype mice) constrained in an identical wired-cage (animal) in the other side chamber. The cylindrical-shaped wired-cages (diameter: 10.5 cm, height: 11.8 cm) were constructed at the precision mechanics facilities of Philipps-University Marburg. The cages had 2 mm thick metal bars spaced 7 mm apart and were closable with a lid. After the social approach trial, the subject mouse was individually kept for 30 min in the previously used Makrolon type III cage (inter-trial interval). Thereafter, subject mice were returned to the three-chambered box for a 10 min social recognition trial. During the social recognition trial, subject mice were given the choice between the stimulus mouse from the previous social approach trial (familiar mouse) in the side chamber where it was presented before or a novel stimulus mouse replacing the empty wired-cage (novel mouse) in the other side chamber. As stimulus mice, age- and sex-matched C57BL/6N mice (Charles River Laboratories, NC, USA) were used. Stimulus mice were group-housed under similar conditions as subject mice and habituated to the wired-cages for 30 min prior testing. Location and stimulus mice presented were counter balanced between subject mice.

#### Novel Object Recognition

After being individually kept for 30 min in a Makrolon type III cage, subject mice were tested for novel object recognition (Bevins and Besheer, 2006), using a modified protocol previously established (Sungur et al., 2017). This test consisted of three phases, i.e., the object acquisition trial (10 min), the inter-trial interval (30 min), and the object recognition trial (10 min). During the object acquisition trial, subject mice were allowed to freely explore for 10 min the three-chambered box containing two identical sample objects, with one sample object being centrally placed in each of the two side chambers. Thereafter, the subject mouse was individually kept for 30 min in the previously used Makrolon type III cage (inter-trial interval). During that time, one of the objects from the object acquisition trial (familiar object) was replaced with a novel object of similar size but different color, shape and material (novel object) to test object recognition memory. Specifically, one clean familiar object and one clean novel object were placed into the three-chambered box, where the two identical objects had been located during the object acquisition trial. After the 30 min delay, each subject mouse was returned to the three-chambered box for a 10 min object recognition trial and allowed to freely explore the familiar and the novel object. As objects, two silver iron cylinders (50 mm in diameter, 80 mm high) and two red metal cubes (50 × 50 × 80 mm) were used. Location and type of objects presented were counter-balanced between subject mice.

### Behavioral Analysis

All behavioral tests were analyzed in videos by an experienced observer blind to the genotype using the Observer XT 10.0 software (Noldus Information Technology, Wageningen, Netherlands). For reciprocal social interaction, parameters of social behaviors included: facial sniffing (sniffing the nose and snout region of the partner), anogenital sniffing (sniffing the anogenital region of the partner), following (walking straight behind the partner, keeping pace with the one ahead), push past (squeezing between the wall and the partner), crawling under/over (pushing the head underneath the partner’s body or crawling over or under the partner’s body), social grooming (grooming the partner), and being socially inactive while having social contact (lying flat or standing still while maintaining close physical contact with the partner), according to previous studies (Terranova and Laviola, 2005; Yang et al., 2012; Wöhr et al., 2015). All social behaviors were analyzed for frequency of occurrence (that is, number of bouts) and duration in 1 min time bins. In addition to social behaviors, non-social behaviors including rearing (number of times an animal reared on its hind legs), grooming (number of bouts of face, body and genital grooming movements) and digging (number of bouts of digging in the bedding, pushing and kicking it around) were counted. For novel object recognition, social approach and social recognition, number of entries into the chambers, the time spent therein, and object investigation were scored. Object investigation was defined as time spent sniffing the social or non-social stimulus when the nose was oriented towards it, with the nose-object distance being 3 cm or less. Novel object recognition and social recognition was defined as spending significantly more time sniffing the novel than the familiar object or mouse, respectively (for details: Sungur et al., 2017).

### Statistical Analysis

For the analysis of direct reciprocal social interaction and the concomitant emission of interaction-induced ultrasonic vocalization (USV), an ANOVA for repeated measurements with the between-subject factor genotype and the within-subject factor test duration was calculated. Novel object recognition, social approach, and social recognition were analyzed using paired *t*-tests for comparing stimuli within genotypes. For novel object recognition and social recognition, behavior recorded in the first 5 min of each trial was included in the statistical analysis, since habituation to novel stimuli is likely to occur in testing periods exceeding 5 min (Bevins and Besheer, 2006). As we expected no sex differences in juvenile mice (Sungur et al., 2017), we pooled male and female mice. Data are presented as mean values ± standard errors of the mean (SEM). A *p*-value of <0.05 was considered significant.

## Results

### Novel Object Recognition Was Impaired in Juvenile Cofilin1 Mutants

**Figure 1:**
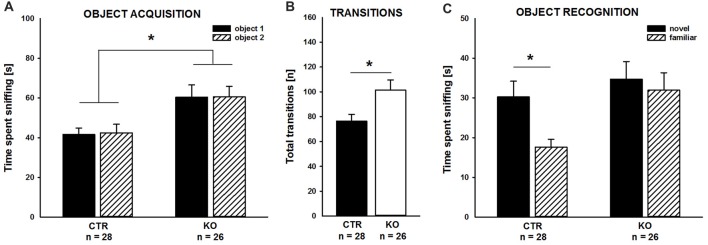
Impaired object recognition in cofilin1 mutant mice. **(A)** Similar to control (CTR), knockout (KO) mice spent equal time exploring both objects during the acquisition phase. Total time exploring the objects was increased in KO mice when compared to CTR mice. **(B)** Activity of KO mice was increased during the acquisition phase as they more often transited between the compartments of the three-chambered box. **(C)** While CTR mice showed a preference for the novel object in the recognition trial, KO mice spent equal time exploring the familiar and the novel object. *p < 0.05.

Our previous studies unraveled deficits in associative learning in adult cofilin1 mutants (Rust et al., 2010). Since we here wanted to study the role of cofilin1 for social behavior in juvenile mice, we tested whether juvenile mutants displayed deficits in non-social memory, similar to adults. To avoid any adverse effect of aversive stimuli during the non-social memory paradigm on social behavior, we chose to perform the novel object recognition test (Bevins and Besheer, 2006). During the object acquisition phase, KO mice spent equal time sniffing the objects in both side chambers, similar to CTR (Figure 1A; CTR: left: 41.6 ± 3.3 s, right: 42.5 ± 4.3 s, *t*_(27)_ = −0.202, *p* = 0.841, *n* = 28 mice (17 males, 11 females); KO: left: 60.4 ± 6.2 s, right: 60.5 ± 5.3 s, *t*_(25)_ = −0.026, *p* = 0.980, *n* = 26 mice (12 males, 14 females)), thereby excluding any side preference which may impede data interpretation. Interestingly, compared to CTR mice, total time of object investigation was increased by more than 40% in KO mice (CTR: 84.1 ± 6.4 s, KO: 120.8 ± 10.3 s, *F*_(1,52)_ = 9.439, *p* = 0.003), and this increase was paralleled by increased numbers of transitions between the chambers (Figure 1B; CTR: 76.3 ± 5.5, KO: 101.4 ± 8.1, *F*_(1,52)_ = 6.725, *p* = 0.012).

As expected, CTR mice preferred the novel to the familiar object during the object recognition trial, and they spent more time sniffing the novel than the familiar object (Figure 1C; novel: 30.2 ± 4.0 s, familiar: 17.6 ± 2.0 s, *t_(27)_* = 2.789, *p* = 0.010). Instead, cofilin1 mutants failed to discriminate between novel and familiar objects, and they spent equal time exploring both objects (Figure 1C; novel: 34.7 ± 4.5 s, familiar: 31.9 ± 4.4 s, *t*_(25)_ = 0.449, *p* = 0.657). The groups also differed in total object exploration time during the recognition trial and KO mice spent more time exploring both objects than CTR (CTR: 47.8 ± 4.4 s, KO: 66.6 ± 6.4 s, *F*_(1,52)_ = 6.043, *p* = 0.017). Together, CTR mice were able to discriminate between novel and familiar objects during the object recognition trial. Instead, KO mice, although spending more time exploring the objects during both the acquisition phase and the recognition trial, showed no preference for novel over familiar objects. These data suggested impaired non-social memory in juvenile cofilin1 mutants.

### Cofilin1 Mutants Showed Normal Social Behavior and Ultrasonic Communication during Direct Reciprocal Social Interaction

**Figure 2:**
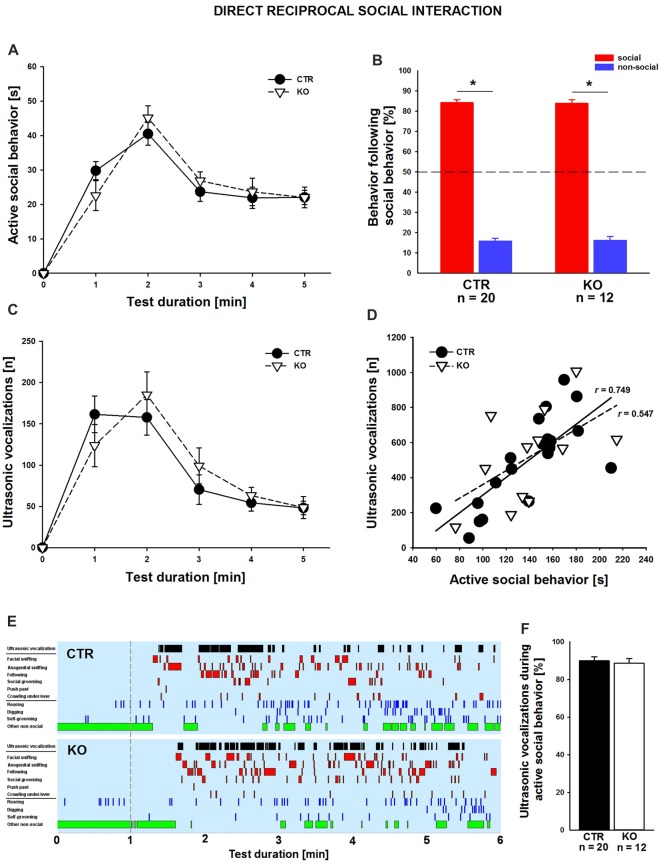
Normal reciprocal social interaction and interaction-induced ultrasonic vocalizations (USVs) in cofilin1 mutants. **(A)** Time spent in active social behavior was similar in CTR and KO mice in 1 min intervals during the 5 min test period. **(B)** The reciprocal character of social activity was similar in CTR and KO mice. In the vast majority of cases, mice of both groups replied with a social behavior to a previous social activity. **(C)** During 5 min of reciprocal social interaction, the number of USV emissions was similar in CTR and KO mice. **(D)** Graph showing the positive correlation of USV emitted during direct reciprocal social interaction and time spent in active social interaction for CTR mice. KO mice showed a trend for this positive correlation, which did not reach statistical significance. **(E)** Representative ethograms of CTR and KO pairs during a 5 min test phase (1–6, min right to the dashed line) upon 1 min of habituation (left to the dashed line). Depicted are ultrasonic vocalizations (black bars in row 1), six different social activities (i.e., facial sniffing, anogenital sniffing, following, social grooming, push past, crawling under/over, red bars in rows 2–7), three different non-social activities (i.e., rearing, digging, self-grooming, blue bars in rows 8–10), and other non-social activities (green bars in row 11). Ethograms revealed that the emissions of USV calls correlate with active social behavior both in CTR and KO mice. **(F)** In CTR and KO mice, the vast majority of USV calls have been emitted during an active social behavior. *p < 0.05.

We next set out to comprehensively characterize social behavior in cofilin1 mutants. We first quantified direct reciprocal social interaction by determining the time mice spent in active social behavior (Terranova and Laviola, 2005; Yang et al., 2012; Wöhr et al., 2015). During the 5 min test period, total time engaging in active social behavior was similar in both groups (CTR: 138.0 ± 8.4 s, *n* = 20 pairs (11 males, 9 females); KO: 140.4 ± 10.7 s *n* = 12 pairs (six males, six females); *F*_(1,30)_ = 0.03; *p* = 0.863), with individual social activities not differing between groups, including facial or anogenital sniffing, following, push past, crawling under/over, or social grooming (data not shown). Likewise, groups did not differ in being socially inactive while having physical contact with the partner (data not shown). Further, no differences between CTR and KO mice were detectable when scoring the time engaging in active social behaviors in 1 min intervals (Figure 2A). We then analyzed the social behavioral repertoire in more detail and found its richness and reciprocal character unaffected in cofilin1 mutants. Specifically, in more than 80% of the cases, both CTR and KO mice preferred to engage in another social behavior following a previous one, not differing from each other (Figure 2B; *F*_(1,30)_ = 0.024, *p* = 0.878). Apart from social behavior, we also analyzed non-social activities, and found no differences between CTR and KO mice when determining total time in non-social behavior (CTR: 95.2 ± 5.9 s, KO: 89.5 ± 8.9 s, *F*_(1,30)_ = 0.010, *p* = 0.920), with individual non-social activities not differing between groups, including rearing, self-grooming or digging (data not shown). Hence, cofilin1 mutants showed no alteration in active social behavior during direct reciprocal social interaction, and they did not display any abnormal repetitive behavior.

Impaired communication has been associated with ASD but also SCZ, and recent studies from us and others reported altered USV emissions for established mouse models (Jamain et al., 2008; Baharnoori et al., 2012; Huang et al., 2014; Lai et al., 2014; Wöhr, 2015; Wöhr et al., 2015; Yang et al., 2015; Kennedy et al., 2016; Sungur et al., 2016). To test whether ultrasonic communication was impaired in cofilin1 mutants, we recorded concomitant emission of interaction-induced USV during direct reciprocal social interaction. Since we only investigated same-genotype pairs, analyses of USV emissions allowed us to judge ultrasonic communication skills in KO mice. When quantifying the total number of USV during the entire test period, we did not find a difference between CTR and KO mice (CTR: 492.7 ± 56.3, KO: 520.0 ± 76.9, *F*_(1,30)_ = 0.084, *p* = 0.773). Moreover, we quantified USV emissions in 1 min intervals and again found no differences between CTR and KO mice (Figure 2C). However, the number of USV emitted during direct reciprocal social interaction was positively correlated with the time spent in active social interaction in CTR but not in KO mice, for which trend was obtained (Figure 2D; CTR: *r* = 0.749, *p* < 0.001; KO: *r* = 0.547, *p* = 0.066). A more detailed temporal analysis revealed that the vast majority of USV (~90%) occurred while mice were engaging in active social behaviors (Figures 2E,F), with the total number of USV emitted during active social behavior not differing between CTR and KO mice (CTR: 436.6 ± 46.9, KO: 465.9 ± 73.1, *F*_(1,30)_ = 0.126, *p* = 0.725). Together, these data exclude any ultrasonic communication deficits during direct reciprocal social interaction in cofilin1 mutant mice.

### Cofilin1 Mutants Showed Normal Social Approach and Recognition Behavior

**Figure 3:**
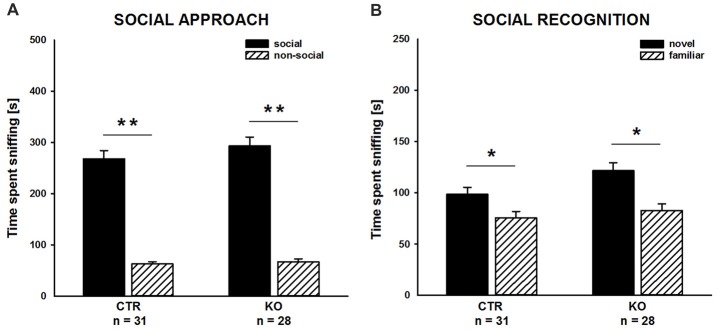
Social approach and social recognition was normal in cofilin1 mutants. **(A)** Similar to CTR, KO mice spent much more time exploring the cage with a social stimulus than the empty cage (non-social). Exploration time of the social and the non-social stimuli was not different between CTR and KO mice. **(B)** CTR and KO mice were both able to discriminate between novel and familiar social stimuli. Both groups spent more time exploring novel than familiar social stimuli. *p < 0.05; **p < 0.001.

We complemented our social behavior analyses by social approach and social recognition tasks to assess social motivation and social cognition, respectively, and, hence, to comprehensively characterize social functioning in cofilin1 mutants. In the social approach paradigm, we tested whether mice preferred a social stimulus to a non-social stimulus. As expected, CTR mice showed a strong preference for the social stimulus, and they spent much more time sniffing the cage with the social stimulus than the empty cage (Figure 3A; social: 268.1 ± 16.3 s, non-social: 62.8 ± 4.7 s, *t*_(30)_ = 11.001, *p* < 0.001, *n* = 31 mice (18 males, 13 females)). Similarly, KO mice showed a very strong preference for the social stimulus (social: 293.6 ± 17.1 s, non-social: 67.4 ± 5.7 s, *t*_(27)_ = 10.496, *p* < 0.001, *n* = 28 mice (13 males, 15 females)), and they spent almost identical time at social and non-social stimuli as CTR mice (*F*_(1,57)_ = 1.175, *p* = 0.283). Moreover, the time exploring the social stimulus was not different between CTR and KO mice (*t*_(57)_ = 1.084, *p* = 0.283). Hence, social approach behavior was unchanged in cofilin1 mutant mice.

To assess social recognition, we tested whether subject mice showed a preference for the familiar or the novel social stimulus. As expected, CTR mice showed a preference for the novel social stimulus, as they spent more time sniffing the cage with the novel mouse than the cage with the familiar one (Figure 3B; familiar: 75.6 ± 6.2 s, novel: 98.5 ± 7.0 s, *t*_(30)_ = 2.375, *p* = 0.024, *n* = 31 mice (18 males, 13 females)). Likewise, KO mice showed a preference for the novel social stimulus. Similar to CTR mice, they spent more time sniffing the novel than the familiar social stimulus (familiar: 82.8 ± 6.5 s, novel: 121.6 ± 7.8 s, *t*_(27)_ = 3.032, *p* = 0.005, *n* = 28 mice (13 males, 15 females)). Of note, compared to CTR mice, KO mice spent more time sniffing the novel social stimulus (*t*_(57)_ = 2.223, *p* = 0.030). Together, our data demonstrated intact social cognition for cofilin1 mutant mice in the three-chambered social recognition paradigm.

## Discussion

In the present study, we investigated specific behavioral domains in juvenile mutant mice lacking cofilin1, a key regulator of actin dynamics with important functions in postsynaptic plasticity of glutamatergic synapses (Rust, 2015; Kanellos and Frame, 2016). While we found a novel object recognition to be impaired in cofilin1 mutants, these mice did not show deficits in a variety of social behaviors or ultrasonic communication. Moreover, they did not show abnormal repetitive behavior. Based on our findings and data from our previous studies (Rust et al., 2010; Goodson et al., 2012; Zimmermann et al., 2015), we conclude that cofilin1 is relevant for non-social cognition, but not for other behavioral domains such as social behavior or ultrasonic communication that are impaired in ASD or SCZ.

To study the relevance of cofilin1-dependent actin dynamics for social behavior and ultrasonic communication, we here exploited juvenile mutant mice lacking cofilin1 in excitatory neurons of the postnatal telencephalon including the prefrontal cortex (PFC, Rust et al., 2010). We decided to perform our experiments in these mutants because ASD-like behavioral abnormalities in juvenile *Shank3* mutant mice were rescued upon acute systemic or PFC-specific inhibition of cofilin1 (Duffney et al., 2015). We previously reported weaker performance of cofilin1 mutant mice in paradigms of associative learning, including spatial learning in Morris water maze, aversive learning in contextual and cued fear conditioning, and rewarded learning in conditioned place preference (Rust et al., 2010). Here, we demonstrated a robust deficit of cofilin1 mutant mice in novel object recognition, as they did not show a preference for the novel over the familiar object in the test trial although spending more time exploring the objects during the acquisition trial. Unlike the previously performed paradigms, novel object recognition is not based on aversive or rewarded stimuli, but takes advantage of the rodents’ intrinsic motivation to approach and explore novelty (Bevins and Besheer, 2006). Hence, cofilin1 is required not only for associative learning, but also for discriminating between novel and familiar objects, which has been termed “non-matching-to-sample” learning (Bevins and Besheer, 2006). Together, our data let us conclude that cofilin1 is relevant for a broad spectrum of cognitive processes. While we previously investigated associative learning in adult mutants (10–16 weeks old), here we report defects in novel object recognition for juvenile mutants (3–4 weeks old). Hence, our data revealed that cofilin1 inactivation in the postnatal telencephalon has immediate consequences for behavior, which is in very good agreement with synaptic deficits that were evident in acute hippocampal slices from 3-weeks-old mutants or in dissociated hippocampal neurons from cofilin1 mutant embryos cultured for 3 weeks (Rust et al., 2010).

Our characterization of social behavior included direct reciprocal social interaction, social approach and social recognition. In none of these paradigms did cofilin1 mutants perform weaker than their control littermates. We therefore concluded that cofilin1 is not relevant for social interaction, social approach or social memory. This was an unexpected finding because cofilin1 has been implicated in social approach and social recognition just recently (Duffney et al., 2015; Foote et al., 2015). Specifically, juvenile mice with a deletion of the ASD-associated gene *Shank3* showed a robust reduction in social approach while other behavioral domains including social or novelty recognition, exploratory behavior, locomotor activity or motor coordination were unaffected. Interestingly, reduced sociability in this specific *Shank3* mouse model was associated with a dysregulation of actin regulatory proteins including an over-activation of cofilin1 and with reduced synaptic F-actin levels, and it was rescued by acute systemic intravenous or PFC-specific injection of a cofilin1-inhibiting peptide (Duffney et al., 2015). Similar to *Shank3* mutant mice, an over-activation of cofilin1 was associated with reduced social approach in 14-3-3 FKO, and these mice displayed additional deficits in social recognition (Foote et al., 2015). However, no rescue experiments with cofilin1-inhibiting peptides have been performed in this study and it therefore remained unknown whether the over-activation of cofilin1 contributed to the SCZ pathology in 14-3-3 FKO. Nevertheless, these studies implicated cofilin1 in social approach and suggested a role for cofilin1 in social recognition, which seemingly is at odds with normal social approach and social recognition in cofilin1 mutants. However, one has to consider that cofilin1 was over-activated in *Shank3* mutants or 14-3-3 FKO, while it was inactivated in our study, and cofilin1 mutants displayed strongly increased synaptic F-actin levels opposite to the reduction reported for *Shank3* mutants (Rust et al., 2010; Duffney et al., 2015; Wolf et al., 2015). Notably, synaptic levels of N-methyl-D-aspartate receptor (NMDAR) subunits were reduced in *Shank3* mutants, and they displayed NMDAR hypofunction (e.g., reduced ratio of NMDAR-to-α-amino-3-hydroxy-5-methyl-4-isoxazolepropionic acid receptor (AMPAR)-mediated currents), which was restored by cofilin1 inhibition (Duffney et al., 2015). The authors therefore speculated that NMDAR hypofunction was the primary pathophysiological cause for the ASD-like phenotype in these *Shank3* mutants, an idea that was in line with several previous studies (Moy et al., 2008; Zou et al., 2008; Carlson, 2012). However, we did not find any evidence for altered NMDAR expression in cofilin1 mutants, and we previously demonstrated a normal ratio of NMDAR-to-AMPAR-mediated currents in these mice (Rust et al., 2010). Hence, normal NMDAR function in cofilin1 mutants could explain different behavioral outcome in *Shank3* and cofilin1 mutant mice. Notably, additional cellular defects have been reported or postulated for other *Shank3* mouse models that also displayed ASD-like behavioral deficits, e.g., impaired cortico-striatal connectivity, altered striatal activity, impaired AMPAR trafficking or altered inhibitory transmission (Peça et al., 2011; Wang et al., 2011; Zhou et al., 2016; Monteiro and Feng, 2017). Such defects might be independent of actin defects and cofilin1 dysregulation and may contribute to the behavioral deficits in *Shank3* mutants reported by Duffney and colleagues.

Apart from social behavior, we also analyzed the emission of interaction-induced USV of juvenile cofilin1 mutant mice during direct reciprocal social interaction to study ultrasonic communication. The analysis of USV emission in behaving mutant mice was inspired by several recent studies that reported alterations in ultrasonic communication in mouse models for ASD or SCZ (Jamain et al., 2008; Baharnoori et al., 2012; Huang et al., 2014; Lai et al., 2014; Wöhr, 2015; Wöhr et al., 2015; Yang et al., 2015; Kennedy et al., 2016; Sungur et al., 2016). In cofilin1 mutants, we did not obtain evidence for prominent deficits in ultrasonic communication, with call rates and temporal emission patterns being similar to control littermates. In line with previous findings (Panksepp et al., 2007), we further showed that the time spent engaging in active social behavior was highly positively correlated with USV emission in control littermates at the inter-individual level. While such a positive correlation did not reach statistical significance in cofilin1 mutants, active social behavior and USV emission were time-locked in both genotypes at the intra-individual level. In fact, in both genotypes ~90% of USV were emitted while engaging in social activities. This finding is in support of the notion that USV emitted during direct reciprocal social interactions serve a pro-social communicative function as social contact calls maintaining social proximity. Together, our data demonstrated that cofilin1 is not relevant for ultrasonic communication in juvenile mice. Hence, two behavioral domains, i.e., social behavior and ultrasonic communication, were not disturbed in cofilin1 mutants, demonstrating that these mice did not display the full spectrum of behavioral symptoms that have been associated with ASD or SCZ.

Apart from the deficit in social approach, juvenile *Shank3* mutant mice also displayed abnormal repetitive behavior as deduced from strongly elevated self-grooming activity of isolated mutant mice when placed in a novel environment (Duffney et al., 2015). Interestingly, acute systemic cofilin1 inactivation normalized self-grooming activity in juvenile *Shank3* mutants, thereby associating dysregulated cofilin1-dependent actin dynamics to abnormal repetitive behaviors. In contrast to *Shank3* mutant mice, juvenile cofilin1 mutants did not show elevated self-grooming activity during reciprocal social interaction. Furthermore, other non-social activities such as rearing or digging were not elevated in cofilin1 mutants during reciprocal social interaction, and we did not observe abnormal repetitive behavior in our previous studies in which we tested individual cofilin1 mutants in novel environments such as an open field arena, Y maze, elevated plus maze or 8-arm radial maze (Rust et al., 2010; Zimmermann et al., 2015). Together, normalization of cofilin1 activity rescued obsessive self-grooming activity in *Shank3* mutants, while inactivation of cofilin1 *per se* did not induce abnormal repetitive behaviors.

In light of the limitations of single-gene knockout approaches, an explanation for the lack of behavioral phenotypes with relevance to human ASD core symptoms in cofilin1 mutant mice could be mechanisms that compensate for the loss of cofilin1. A good candidate for that is ADF, a close cofilin1 homolog with similar biochemical functions, which is present at excitatory synapses (Görlich et al., 2011). Indeed, double mutant mice lacking cofilin1 and ADF displayed behavioral deficits such as hyperactivity, impaired working memory or a paradoxical calming effect of psychostimulants that were not present in single mutants (Zimmermann et al., 2015), thereby demonstrating redundant functions for cofilin1 and ADF in behavior. However, social behavior has not been tested in these mutants. Moreover, considering the known heterogeneity of the etiology and symptomology of ASD, experimental manipulations of genetic background and environmental conditions appear warranted when assessing the role of cofilin1 in regulating social behavior in future studies.

In summary, we here report defects in novel object recognition that corroborated cognitive impairments in cofilin1 mutant mice (Rust et al., 2010). We previously reported that cognitive decline in cofilin1 mutants was associated with a reduction of anxiety-related behavior and a moderate increase in locomotion, while other behavior domains including working memory, nest-building, impulsivity or response to psychoactive drugs were unchanged (Rust et al., 2010; Goodson et al., 2012; Zimmermann et al., 2015). We confirmed increased locomotion in the present study by the elevated number of transitions during novel object recognition. Instead, we reported here normal social behavior, normal ultrasonic communication and the absence of abnormal repetitive behavior in juvenile cofilin1 mutants. Hence, cofilin1 mutants showed deficits specifically in non-social cognitive tasks, but they did not display an ASD- or a SCZ-like phenotype.

## Author Contributions

MW, MBR and AÖS designed the research and wrote the manuscript. AÖS and LS performed the research.

## Conflict of Interest Statement

The authors declare that the research was conducted in the absence of any commercial or financial relationships that could be construed as a potential conflict of interest.

## Abbreviations

ADF: actin depolymerizing factor
AMPA: α-amino-3-hydroxy-5-methyl-4-isoxazolepropionic acid
AMPAR: AMPA receptor
ASD: autism-spectrum disorder
CaMKII: CaM-kinase II
CTR: control
F-actin: actin filament
FKO: functional knockout
ID: intellectual disability
KO: knockout
NMDA: N-methyl-D-aspartate
NMDAR: NMDA receptor
PFC: prefrontal cortex
PND: postnatal day
SCZ: schizophrenia
USV: ultrasonic vocalization.
